# Attrition and discontinuation in amyotrophic lateral sclerosis clinical trials: a meta-analysis

**DOI:** 10.1007/s00415-024-12813-3

**Published:** 2024-12-12

**Authors:** Ruben P. A. van Eijk, Floris T. van Loon, Jordi W. J. van Unnik, Daphne N. Weemering, Georgios Seitidis, Dimitris Mavridis, Leonard H. van den Berg, Stavros Nikolakopoulos

**Affiliations:** 1 Department of Neurology, UMC Utrecht Brain Center, University Medical Center Utrecht, Heidelberglaan 100, 3584 CX Utrecht, The Netherlands; 2 Biostatistics and Research Support, Julius Center for Health Sciences and Primary Care, University Medical Center Utrecht, Utrecht, The Netherlands; 3 Department of Psychology, University of Ioannina, Ioannina, Greece; 4 Department of Primary Education, School of Education, University of Ioannina, Ioannina, Greece

**Keywords:** Treatment discontinuation, Attrition, Clinical trials, Meta-analysis, ALS

## Abstract

**Objectives:**

Attrition due to adverse events and disease progression impacts the integrity and generalizability of clinical trials. The aim of this study is to provide evidence-based estimates of attrition for clinical trials in amyotrophic lateral sclerosis (ALS), and identify study-related predictors, through a comprehensive systematic review and meta-analysis.

**Methods:**

We systematically reviewed the literature to identify all randomized, placebo-controlled clinical trials in ALS and determined the number of patients who discontinued the study per randomized arm. Subsequently, we meta-analyzed attrition rates across studies, evaluated the difference between study arms, and explored the impact of study-level characteristics. Finally, a meta-regression model predicting study discontinuation for future clinical trials was translated into a web application.

**Results:**

In total, 60 randomized placebo-controlled clinical trials were included in the meta-analysis, randomizing 14,493 patients with ALS. Attrition varied significantly between studies, ranging from 3.1% to 75.7% of all randomized patients, with a pooled effect of 32.0% (90% prediction interval 6.1% to 66.3%). Attrition was similar between the intervention and placebo arm (odds ratio 1.08, 95% CI 0.89 to 1.31, *p* = 0.43). The follow-up duration was identified as the sole study-level predictor (0.032, 95% CI 0.026 to 0.039, *p* < 0.001), resulting in predicted attrition of 19.3% for 6-month, 36.4% for 12-month, and 55.6% for 18-month clinical trials.

**Conclusions:**

ALS clinical trials encounter high attrition, which increases with the follow-up duration. These findings underscore the need to refine our strategies to manage attrition, preserving the integrity and generalizability of ALS clinical trials.

## Introduction

Attrition and study discontinuation pose significant challenges in clinical trials for Amyotrophic Lateral Sclerosis (ALS) [1]. These issues affect the integrity of a clinical trial [2], diminishing its precision due to a loss in statistical power and potentially introducing bias. Bias may occur if patients who (decide to) discontinue differ systematically from those who remain in the study, or if attrition varies between randomized arms [3, 4]. As study discontinuation occurs after randomization, it distorts the balance that was initially created and directly influences the estimates of the treatment effect. This potentially compromises the trial's internal validity and may question the observed impact of the intervention on study outcomes [5, 6]. Extensive attrition may also hinder the external validity and generalizability of study findings, raising concerns about the applicability of the results to broader patient populations, or remaining ambiguous regarding to whom the study results still apply [7].

Nevertheless, despite the substantial impact of study discontinuations on trial integrity, the issue has received little attention in the ALS literature. The rapidly progressive nature of ALS, however, coupled with its high mortality rate, has led to non-trivial attrition in ALS clinical trials [1, 8, 9]. Attrition thus poses a substantial challenge, though accurate estimates of the expected attrition rate are currently not available. Moreover, the patient community has expressed a desire for more patient-focused clinical trials [10], e.g., by using more frequently unequal randomization ratios and open-label extensions. Gaining insight into how these trial design features influence study discontinuation, or having a more accurate estimate of the expected study attrition, could help improve the design and conduct of future clinical trials.

In this study, therefore, we aim to address the current gaps in the literature by conducting a comprehensive systematic review and meta-analysis, thereby providing evidence-based estimates of attrition in ALS clinical trials and identifying potential study-related contributors. The results are presented in an online web application to support the design of future clinical trials.

## Methods

This study consisted of three parts. First, we systematically reviewed the literature to identify all randomized, placebo-controlled clinical trials in ALS and determine the number of patients who discontinued the study per randomized arm (i.e., placebo vs. active). Subsequently, we meta-analyzed discontinuation rates per study arm and explored the impact of study-related factors on the discontinuation rate. Finally, we translated results into an open-access web application for clinical trial design.

### Search strategy and study selection

The protocol of the search strategy has been described elsewhere [11]. In brief, two reviewers individually searched the PubMed and Embase database for publications dating from the first of January 2000 up to and including November 2023, using the following search terms: “amyotrophic lateral sclerosis” or “motor neuron disease” and “clinical trial*”. To harmonize the comparison between clinical trials, we only included randomized, placebo-controlled clinical trials evaluating the efficacy of a single pharmacological agent. We excluded clinical trials investigating multiple agents, exclusively aiming to determine safety (phase I), having a non-clinical primary endpoint, starting enrollment before the approval of Riluzole (1996), or having a sample size of less than 15 patients per arm.

### Data extraction and definitions

From each study, we extracted the number of patients in the active and placebo groups who discontinued the study, together with the reason (categorized as death, adverse event, or other). Patients who encountered two reasons for discontinuation were classified according to the first reason for discontinuation. For example, a patient who discontinued due to an adverse event and subsequently died, was counted as a discontinuation due to adverse event and not due to death. For four studies it was unclear how many deaths occurred among the discontinuations for other reasons; for these studies, we assumed none of the deaths was (doubly) reported among the other discontinuations. The number of patients in each randomized arm (i.e., the denominator of the discontinuation rate) was based on all randomized patients. Patients who were discontinued early due to a misdiagnosis were excluded. For the Tirasemtiv study specifically [12], as a randomization error led to a study drug assignment error (excluding 156 patients of the 605 randomized), we assumed a randomized sample size of 449 (i.e., 605–156).

In addition, we extracted study-level characteristics that may have affected study discontinuation including year of first enrolled patient, sponsor type (academic or industry), route of treatment administration (oral or other), randomization ratio (equal or unbalanced), use of a lead-in period, open-label access at the end of the double-blind period, number of in-clinic visits per month, and the follow-up time. The follow-up time of each study was based on the reported study design in case of a fixed duration design (i.e., all randomized patients are followed for a fixed duration). For studies with extended or variable randomized follow-up (e.g., patients enrolled early have a longer follow-up than patients enrolled later), or when the study was stopped early at an interim analysis [13], we took either the reported average patient follow-up time, calculated the average follow-up time based on the number at risk table, or estimated an average by assuming a uniform enrollment rate and a minimum follow-up time after the last enrolled patient [14]. Follow-up time was counted only during the randomized, placebo-controlled period; discontinuations occurring during an open-label period were excluded.

### Statistical analysis

The primary objective of the analysis was to estimate the proportion of patients who discontinued the study, defined as the number of study discontinuations among all randomized patients. For each study, per randomized arm, we calculated the arcsine square root transformed proportion of the treatment discontinuation rate [15]. A transformed proportion was calculated in order to stabilize study variances, which benefits studies with few or substantial discontinuations (i.e., proportions close to zero or one) [16]. The transformed proportions were then pooled across randomized arms within each study by fixed-effects meta-analysis, and subsequently pooled across studies by random-effects meta-analysis using Restricted Maximum Likelihood (REML) estimation. Meta-analysis estimates were back-transformed and presented on the original scale, together with a 95% confidence interval (CI), 90% prediction interval, the results from the Q test for between-study variability and the *I*^2^ statistic.

As a secondary objective, we assessed whether study discontinuations were different between treatment arms, i.e., differential attrition. For each study, we determined the proportion of patients who discontinued in the intervention and placebo arms based on a 2 by 2 table. Subsequently, we calculated the odds ratio so that a ratio greater than one suggests higher discontinuation rate in the intervention group, whereas a ratio less than one suggests higher discontinuation rate in the placebo group. Studies with no discontinuations in either the intervention or the placebo arm were handled by adding 0.50 to each cell in the 2 by 2 table [4, 5]. Study results were pooled by random-effects meta-analysis using Restricted Maximum Likelihood (REML) estimation. The primary and secondary analyses were repeated for the different components of study discontinuation, namely “adverse events”, “death” and “other reasons”. We also calculated non-death-related attrition, defined as (# of discontinuations – # of deaths) / (# of randomized patients – # of deaths).

Finally, a mixed effects meta-regression model was used to evaluate which study-level characteristics were associated with the degree of study discontinuation. A single multivariable mixed effects meta-regression model was constructed including all study-level characteristics listed above. The final meta-regressive model, including only the statistically significant study characteristics, was used to estimate prediction intervals for future clinical trials. For continuous covariates, we also explored adding a quadratic term to the model. We evaluated how much between-study variability was explained by the final selected set of predictors by calculating the reduction in $\tau^{2}$ (between-study variability) compared to a null model. Confidence intervals around the reduction in $\tau^{2}$ were obtained by means of bootstrapping (500 iterations). Predictions were translated into a Shiny web application.

In some studies, reporting of study discontinuations was incompletely; as a result, data on either the total number of study discontinuations per randomized arm or the reasons for discontinuation were missing. For these studies, missing data were accounted for by creating multiple imputed datasets (*n* = 100) using predictive mean matching, thereby disregarding the first 100 iterations (burn-in). The imputation model contained all components of study discontinuation and the study-level characteristics. Imputed values were constrained by the sample size of each arm or by known total or sub-total study discontinuations. Subsequently, the analysis was conducted in each imputed dataset; results across imputed datasets were summarized using Rubin’s rules [17]. As sensitivity analysis, we repeated the analysis on the subset of studies with complete data [18]. All analyses were conducted in R using the *metafor* library (Viechtbauer W, version 4.4-0, 2023) [19].

## Results

**Fig. 1:**
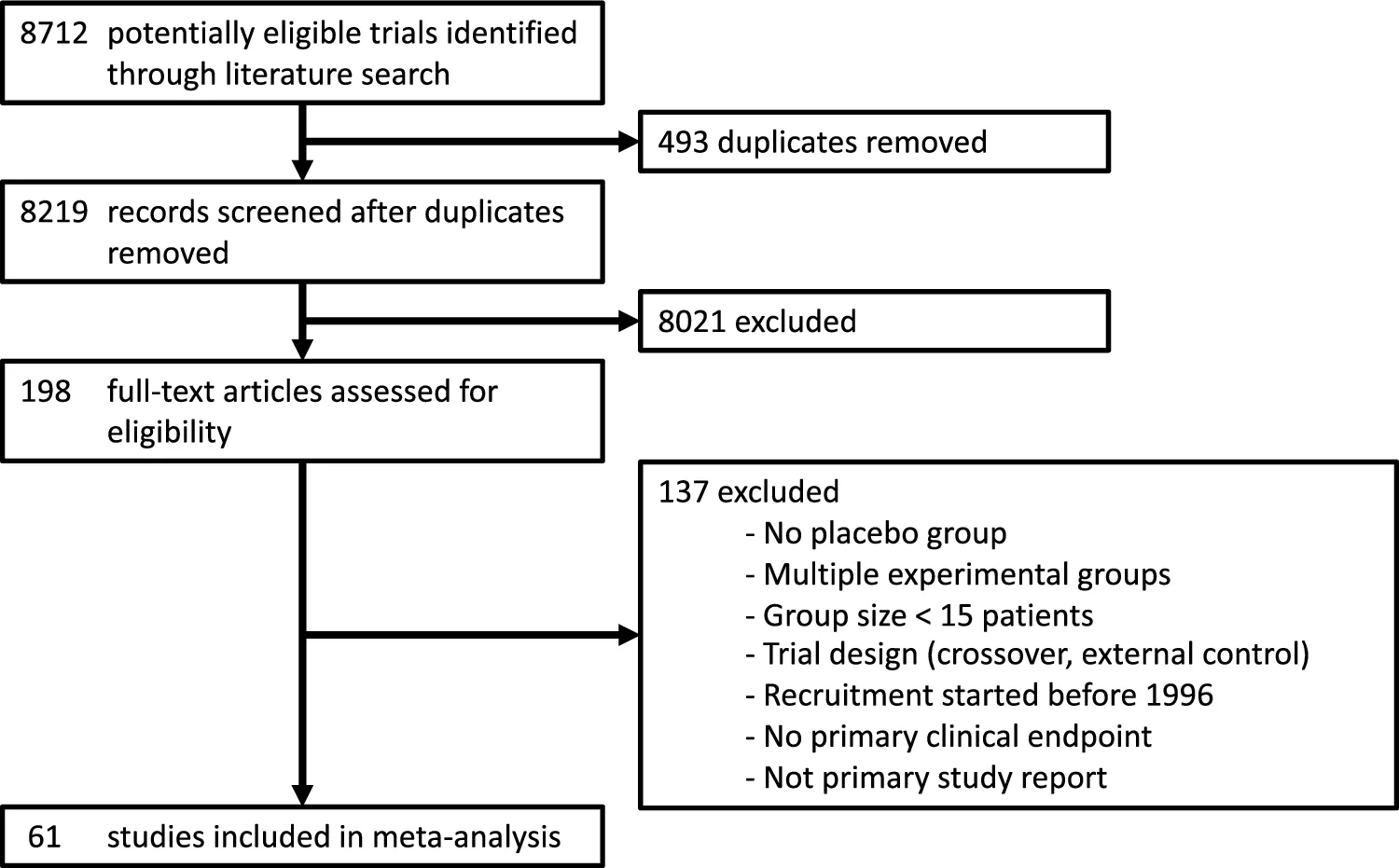
Flow diagram of study selection. Flow diagram of the systematic search and identification of randomized, placebo-controlled

A total of 61 randomized placebo-controlled clinical trials were identified (Fig. 1). One study was excluded from the final analysis as the total number of discontinuations could not be determined [20]. The study characteristics of the remaining 60 studies are presented in Table 1, resulting in a total sample size of 14,493 patients with ALS. The median sample size of the included studies was 155 patients with a range of 32 to 2,077 patients (interquartile range 77 to 305). Nine studies (15%) did not report discontinuations per randomized arm and/or the reason for discontinuation; missing data in these studies were addressed by multiple imputation.

**Table 1:** Overview of included randomized placebo-controlled clinical trials

Study characteristic	All clinical trials (*N* = 60)
Total number of randomized patients	14,493
Active	8451 (58%)
Placebo	6042 (42%)
Median sample size (range)	155 (32–2077*)
Median follow-up duration in months (range)	11.0 (2.8–24.0)
Equal randomization ratio*, yes*	42 (70%)
Administration route	
Oral	44 (73%)
Intravenous	11 (18%)
Intramuscular	2 (3%)
Intrathecal	1 (2%)
Subcutaneous	2 (3%)
Open-label extension (yes)	15 (25%)
Lead-in period (yes)	11 (18%)
Study sponsor (academic)	37 (62%)

^*^ The xaliproden study was pooled across both arms, resulting in a pooled sample size of 2077

### Study discontinuation

**Fig. 2:**
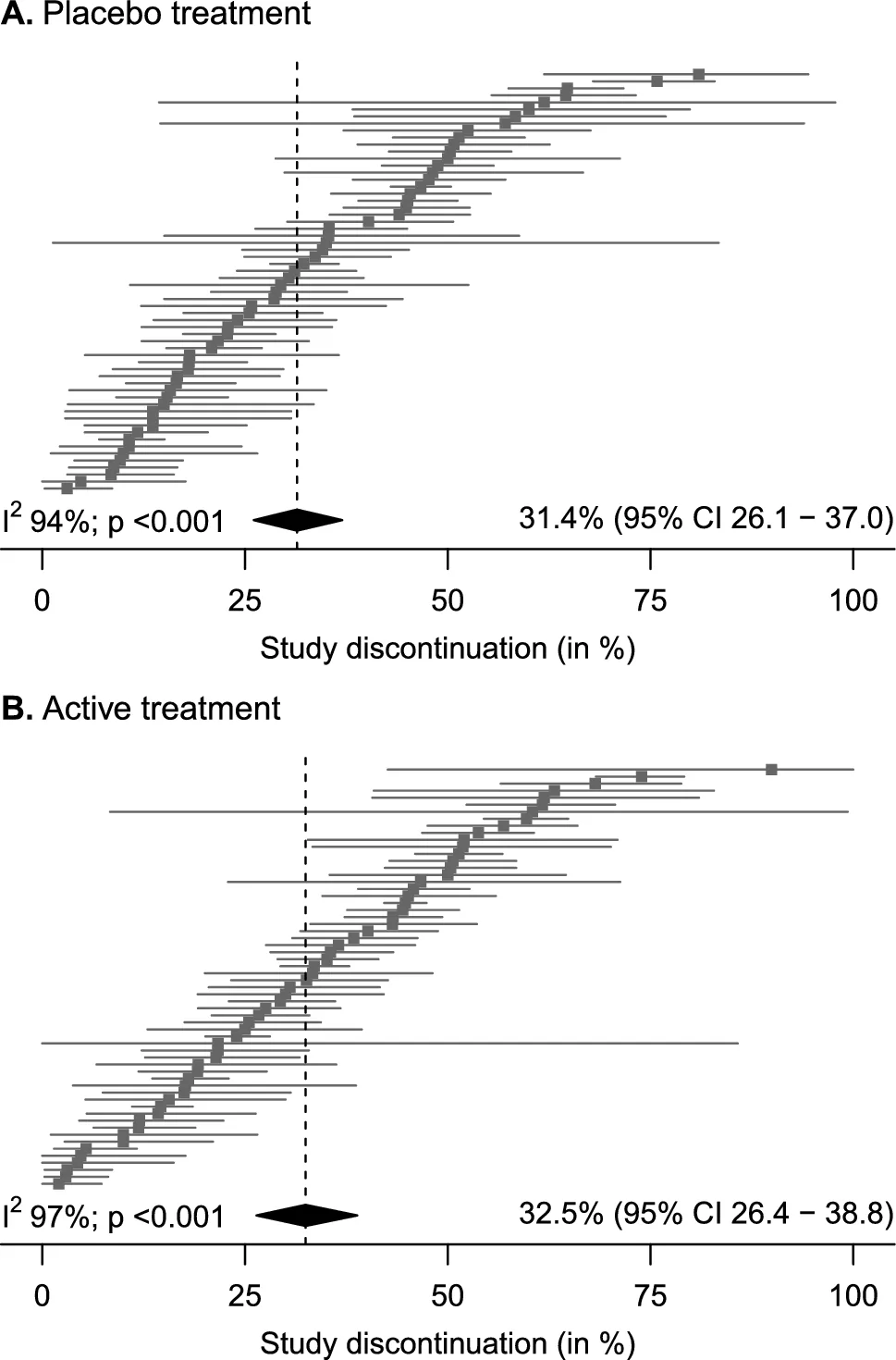
Between-study variability in discontinuation rates among ALS clinical trials. Caterpillar plot of study discontinuation among randomized patients. For each study (N = 60), the total number of study discontinuations was extracted for the placebo (**A**) and active (**B**) treatment arm. Solid black diamonds reflect the meta-analyzed average proportion of study discontinuations, together with its 95% confidence interval. Extensive study heterogeneity was present in both the placebo (*I*^2^ = 94%) and active arm (*I*^2^ = 97%)

There was significant between-study heterogeneity in discontinuation rates between the 60 studies (*I*^2^ 97.7%, Cochran’s *Q* test* p* < 0.001, $\tau^{2}$ 0.0447, Fig. 2), with attrition ranging from 3.1% to 75.7% of the randomized patients. The meta-analyzed average discontinuation rate across studies was 32.0% (95% CI 26.8% to 37.4%; 90% prediction interval 6.1% to 66.3%). Discontinuation rates were numerically lower in the placebo arm compared to the intervention arm (31.4% vs. 32.5%), though there was no statistical evidence for differential discontinuation rates between arms (OR 1.08, 95% CI 0.89 to 1.31, *p* = 0.43). There was, however, large variability between studies in ORs (*I*^2^ 64.0%, Cochran’s *Q* test* p* < 0.001). In total, 8 studies reported an OR ≥ 2.0, meaning the odds to discontinue in the treatment arm were twice the odds in the placebo arm, whereas 10 studies reported an OR ≤ 0.5.

The different reasons for study discontinuation are presented in Table 2. Overall, discontinuation due to ‘other reasons’, including lost-to-follow-up and disease progression, was the major contributor to discontinuation followed by ‘death’. The meta-analyzed average non-death-related attrition was 23.8% (95% CI 19.4% to 28.5%). Discontinuation due to ‘adverse events’ played a relatively minor role. The odds to discontinue the study due to adverse events was, however, 63% higher for patients who had received an active treatment compared to the odds of patients who had received a placebo (OR 1.63; 95% CI 1.22–2.18, *p* = 0.002). Results were similar when the nine studies with missing data were excluded (OR 1.69; 95% CI 1.39–2.05, *p* < 0.001).

**Table 2:** Different components of attrition and study discontinuation stratified by treatment arm

Parameter	Discontinuation rate (95% CI)			Differential attrition (intervention vs. placebo)			
	All patients	Placebo	Intervention	Odds ratio	95% CI	*P* value	*I*^2^
Any discontinuation	32.0% (26.8–37.4)	31.4% (26.1–37.0)	32.5% (26.4–38.8)	1.08	0.89–1.31	0.430	64.0%
Adverse events	5.6% (4.3–7.1)	4.3% (3.1–5.7)	6.7% (4.8–8.8)	1.63	1.22–2.18	0.002	41.8%
Other reasons	13.8% (11.1–16.8)	14.5% (11.5–17.8)	13.4% (10.2–16.9)	0.93	0.74–1.17	0.520	59.0%
Death	9.3% (6.9–12.0)	9.8% (7.2–12.7)	9.0% (6.6–11.8)	0.94	0.86–1.02	0.136	0.0%
Non-death discontinuation	23.8% (19.4–28.5)	22.7% (18.1–27.7)	24.7% (19.2–30.7)	1.13	0.89–1.43	0.327	68.0%

Discontinuation rates are expressed as percentage of the randomized sample size. Note: The percentages are extracted from separate meta-analysis models; although ‘any discontinuation’ is the sum of ‘adverse events’, ‘other reasons’ and ‘death’, the estimated proportions don’t necessarily add up as components were analyzed in separate random effects meta-analysis models. Differential attrition was determined by first calculating an odds ratio for each study (proportion attrition intervention vs. placebo), which were subsequently pooled in a random effects meta-analysis model

### Predictors of study discontinuation

The impact of study-level characteristics on the study discontinuation rate are presented in Table 3. A difference in follow-up duration was the main predictor of attrition (*p* < 0.001); other study-level characteristics were not statistically significantly associated with the discontinuation rate. A meta-regression model with follow-up duration as sole predictor explained 69.0% (95% CI 54.0 to 80.2) of the between-study variation, reducing between-study variability ($\tau^{2}$) from 0.0447 to 0.0139. Nevertheless, after accounting for the follow-up duration, residual between-study variability remained (Cochran’s *Q* test, *p* < 0.001). Adding a non-linear (quadratic) term for follow-up duration did not substantially improve model fit (*p* = 0.83).

**Table 3:** Predictors of study discontinuation in ALS clinical trials

Model predictor	Coefficient	95% CI	*P* value
Intercept	0.2858	0.100 to 0.471	–
Follow-up duration, *months*	0.0309	0.023 to 0.039	< 0.001
Academic sponsor	− 0.0068	− 0.081 to 0.068	0.85
Use of lead-in period	− 0.0658	− 0.155 to 0.023	0.1455
Unbalanced randomization ratio	0.0505	− 0.023 to 0.124	0.1769
Oral route of administration	0.0335	− 0.046 to 0.113	0.40
Availability of open-label extension	− 0.0592	− 0.141 to 0.023	0.1522
Number of in-clinic visits per month (visits per months)	− 0.0292	− 0.105 to 0.046	0.44
Year of first enrolled patient (years since 2000)	0.0003	− 0.005 to 0.006	0.91

Multivariable meta-regression model of the proportion of study discontinuations as function of different covariates. Due to the arcsine square root transformation, the model coefficients are difficult to interpret; no straightforward transformation exists. In general, a positive coefficient reflects an increasing study discontinuation proportion and a negative coefficient a decreasing proportion

**Fig. 3:**
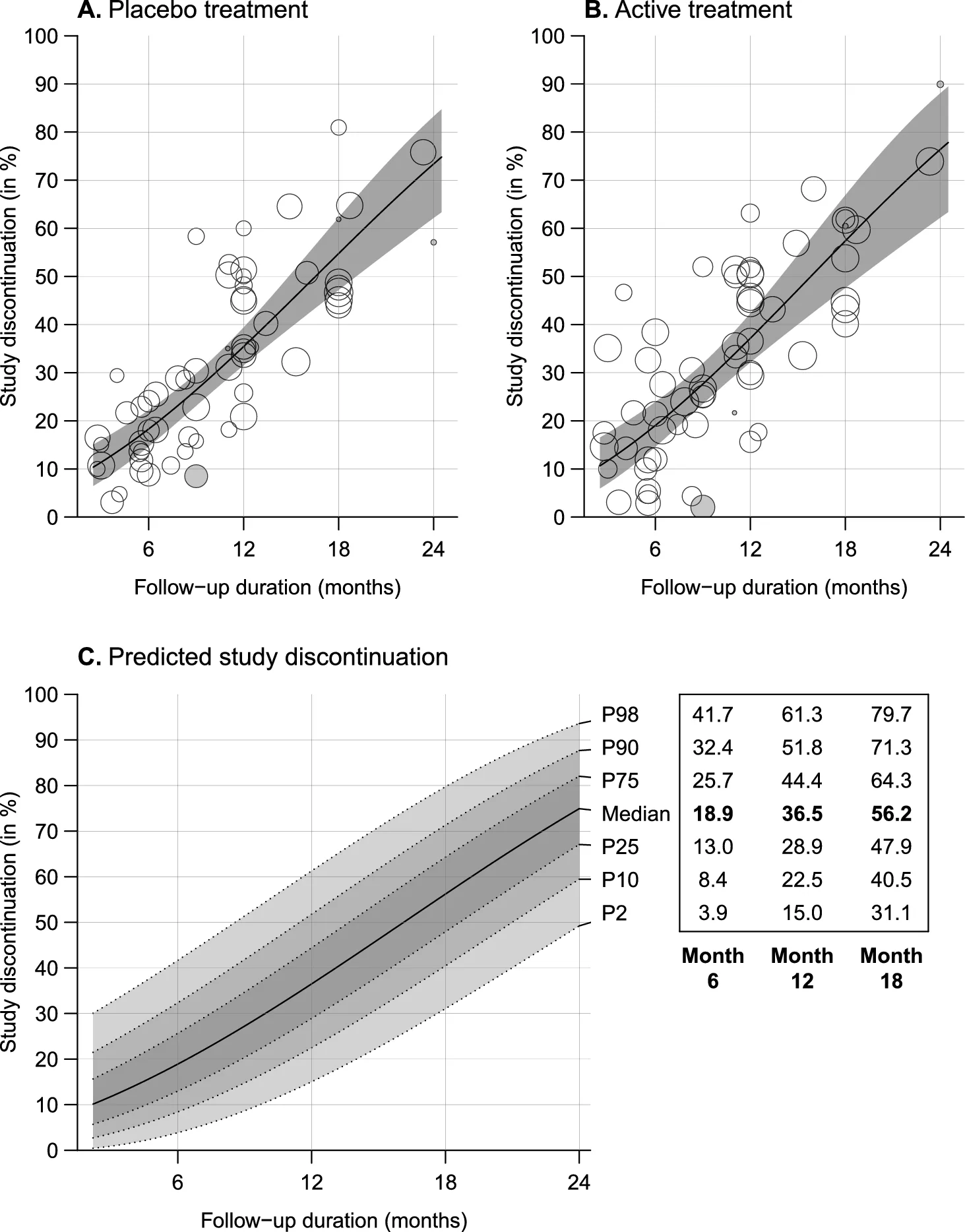
Derivation of predicted study discontinuation rate during follow-up. Meta-regressive model with follow-up duration as single predictor. Dots reflect individual studies, their size are relative to the model weight; gray shaded area is the confidence interval; blue dots are imputed study data (averaged over 100 imputations) in placebo (**A**) and active (**B**) treated groups. **C** The model output and residual between-study variability were used to derive a normogram; the dashed lines reflect the different percentiles: 2nd (P2), 10th (P10), 25th (P25), 75th (P75), 90th (P90) and 98th (P98)

The final meta-regressive model with follow-up duration as sole predictor was used to estimate the study discontinuation for future ALS clinical trials; the coefficient for follow-up time in this model was 0.032 (95% CI 0.026 to 0.039). The model output—including the residual (unexplained) between-study variability—was translated to a normogram (Fig. 3C). The normogram provides a probability range, or prediction interval, for study discontinuation given a study’s follow-up duration for future studies that are similar to those included in the meta-analysis. For example, the expected discontinuation rate for a 12-month study is 36.5% of all randomized patients, with a 10% probability that it will be less than 22.5% (i.e., 10th percentile or P10) or more than 51.8% (i.e., 90th percentile or P90). An online web application was developed to provide the exact prediction interval for different follow-up durations, and to assess the likelihood of observing a certain discontinuation rate (https://tricals.shinyapps.io/attrition/).

## Discussion

In this study, we show that there is large variability in the number of patients in placebo-controlled clinical trials for ALS who will stop the study prematurely, with an average of 32.0% of the randomized patients. This results in significant missing data, loss of precision and, potentially, bias in study outcomes. Attrition is strongly related to the study duration, with predicted rates ranging from 18.9% of the randomized patients at 6 months to 56.2% at 18 months. Thus, even in relatively short studies, high attrition rates are likely, and could hinder the external validity and generalizability of study outcomes. These findings, therefore, underscore the need to refine operational and methodological strategies to better manage attrition, and preserve the integrity and generalizability of ALS clinical trials.

A key consideration is that the extent and impact of attrition in a clinical trial are largely driven by the study’s primary outcome. In our study, attrition included patient death as one of the main determinants, making the estimated discontinuation rates most applicable to clinical trials with intermediate primary outcomes, such as measures of daily functioning or quality of life. However, in clinical trials where overall mortality is the primary outcome, death is no longer considered attrition, which may benefit the external validity and generalizability of the study outcomes. Since patient death remains, unfortunately, an expected event in ALS drug development, especially in studies with prolonged follow-up, it could be beneficial to consider a primary outcome based solely on survival time or one that includes mortality in a composite framework.

Regardless of the main determinants of attrition, and despite the changing clinical trial landscape and increasing advocacy for patient-focused design aspects [10], including open-label extensions and unbalanced randomization ratios, attrition rates in ALS clinical trials have been stable over the last few decades. An earlier study found nearly identical estimates with a mean attrition rate of 33% and a non-death attrition rate of 22% in ALS clinical trials published between 1979 and 2011 [1]. This trend was confirmed in our meta-regression model, which failed to detect a relationship between attrition and the year of enrollment. Moreover, we could not identify an evident association between attrition and allocation ratio, or the availability of an open-label extension at the end of the placebo-controlled period. The duration of follow-up was the only study-level factor that was strongly related to attrition.

Nevertheless, after accounting for a difference in follow-up duration, significant study heterogeneity remained. One potential cause for the residual study heterogeneity is a difference in reporting accuracy and the definition of attrition itself. An example is a patient discontinuing treatment, but continuing follow-up for the primary outcomes. In this setting, authors could provide in this setting the number of patients who discontinued treatment *or* the number of patients who discontinued follow-up; a non-trivial detail, which unfortunately could not always be distilled from the study report. Standardizing the definition and reporting of attrition in ALS clinical trials, therefore, could improve between-study comparability.

Another contributing factor to between-study heterogeneity is likely related to issues inherent to the study drug itself. Our results indicated that the odds of discontinuing the study due to adverse events were significantly higher for patients who received an active treatment compared to those who received a placebo. Although adverse events led, on average, to study discontinuation in only 5.6% of patients, in some clinical trials this was a substantially higher proportion. For example, in studies with perampanel and lithium, over 20% of patients randomized to the active arm discontinued due to adverse events, which will contribute to between-study variability.

Differences between enrolled patient populations likely further drive study heterogeneity in attrition rates. Disease severity, progression rate, and a poor prognosis have been linked to study retention [1, 8, 9], and are factors known to be associated with overall survival [21]. Prognostic diversity in enrolled populations, for example as a result of different sets of eligibility criteria or recruitment strategies [22], could, therefore, impact attrition and result in between-study variability. As these factors relate to the patient, a study-level meta-analysis with aggregate data is not suitable for investigating the impact of patient characteristics without a potential for ecological fallacy (Simpson’s paradox) [23].

Hence, there is a need for individual participant data from clinical trials to obtain a better understanding of the patient-level mechanisms related to attrition. An example was recently provided for MND–SMART [8], but larger scale initiatives are needed to generalize results globally. Such data may also be used to derive patient-level prediction rules for study retention to better inform strategies that prevent attrition in future clinical trials—which, as yet, have not been developed for ALS. Dedicated qualitative studies to derive prognostic patient-level attributes [24], and study-level (or site-level) characteristics [25], could be an important first step in predicting attrition.

It should be noted, however, that some level of attrition will be unavoidable, simply due to the progressive and fatal nature of ALS. Attrition strategies should be focused, therefore, on minimizing attrition, facilitating long-term data collection and should have a pre-specified plan for the statistical analysis. If a patient can no longer visit the clinic, decentralized monitoring could be considered with safety assessments conducted locally by the general practitioner, allowing the patient to continue study medication [26]. If this is not feasible, investigators can provide the means—e.g., using remote technology or home-nursing—to continue data collection at the patient’s home for the main study outcomes (e.g., ALSFRS-R and survival).

The inclusion of data obtained after discontinuation of the study medication may, however, dilute the treatment effect. This should be accounted for in the sample size at the design stage; the online tool can be used to make informative assumptions. Finally, one might consider defining attrition as a clinical milestone given its strong relationships with disease severity and survival. Together with other clinical milestones, such as death, respiratory insufficiency or care dependency, the inability to participate may be integrated as a composite endpoint that could serve as primary efficacy endpoint. As not all milestones would have the same clinical significance, e.g., death is worse than the inability to participate, an outcome based on win statistics could be considered [27, 28].

In conclusion, our results reveal that a non-trivial proportion of the randomized patients in placebo-controlled clinical trials for ALS will stop the study prematurely, even when study duration is limited. Preventive strategies are indicated, but it should be noted that some level of attrition will be unavoidable in ALS clinical trials. It is recommended, therefore, that clinical trials align the definition and reporting of attrition, include elements of decentralized or home monitoring in the study protocol, account for the expected attrition at the design stage, and consider the inability to continue the study as a potential clinical milestone that is integrated with the primary efficacy outcome.

## Data Availability

Data is available at https://tricals.shinyapps.io/attrition/
